# Co-existence of NDM-1 and OXA-48 genes in Carbapenem Resistant *Klebsiella pneumoniae* clinical isolates in Kafrelsheikh, Egypt

**DOI:** 10.4314/ahs.v21i2.2

**Published:** 2021-06

**Authors:** Ramadan Ahmed El-Domany, Tarek El-Banna, Fatma Sonbol, Samar Hamed Abu-Sayedahmed

**Affiliations:** 1 Department of Microbiology and Immunology, Faculty of Pharmacy, Kafrelsheikh University, Kafrelsheikh, Egypt. ramadaneldomany@yahoo.com; 2 Department of Pharmaceutical Microbiology, Faculty of Pharmacy, Tanta University, Tanta, Egypt. t_elbanna@yahoo.com; 3 Department of Pharmaceutical Microbiology, Faculty of Pharmacy, Tanta University, Tanta, Egypt. f_sonbol@yahoo.com; 4 Department of Microbiology and Immunology, Faculty of Pharmacy, Kafrelsheikh University, Kafrelsheikh, Egypt. samarhamed580@yahoo.com

**Keywords:** *Klebsiella pneumoniae*, Egypt, carbapenem resistance, MDR, PCR, blaNDM-1, blaOXA-48, sequencing

## Abstract

**Background:**

The noteworthy spread of carbapenem-resistant *K. pneumoniae* (CR-KP) isolates represents a significant safety threat.

**Objective:**

Determination of the carbapenemase genes incidence among CR-KP clinical isolates in Kafrelsheikh, Egypt.

**Methods:**

A total of 230 *K. pneumoniae* isolates were recovered from four hospitals in Kafrelsheikh, Egypt. Susceptibility testing was conducted using Kirby-Bauer method and automated-Vitek2 system. CR-KP isolates were tested using modified Hodge test (MHT) and combined disk synergy test. PCR and DNA sequencing were conducted for CR-KP isolates to recognize the included carbapenemase-genes.

**Results:**

Out of 230 *K. pneumoniae* isolates, 50 isolates presented resistance to carbapenem (meropenem). All 50 CR-KP isolates were multidrug-resistant (MDR). Genes like blaNDM-1 and blaOXA-48 were the only detected genes among CR-KP with an incidence of 70.0% and 52.0%, respectively. Up to 74.0% of the tested isolates carried at least one of the two recorded genes, among them 48.0% co-harbored both blaNDM-1 and blaOXA-48 genes. The accession-numbers of sequenced blaNDM-1 and blaOXA-48 genes were MG594615 and MG594616, respectively.

**Conclusion:**

This study reported a high incidence of MDR profile with the emergence of blaNDM-1 and blaOXA-48 genes co-existence in CR-KP isolates in Kafrelsheikh, Egypt. Hence, more restrictions should be applied against the spread of such serious pathogens.

## Introduction

*Klebsiella pneumoniae* (*K. pneumoniae*) is a member in *Enterobacteriaceae* family causing serious opportunistic hospital and community-associated infections^1^–^3^. It is known as one of the most common MDR pathogens showing resistance to different classes of antibiotics additionally with multiple mechanisms of antibiotic resistance due to easily acquisition of drug resistance genes through transferable elements (plasmids and transposons) 4–6. Moreover, the infections caused by those serious pathogens are often concomitant with prolonged hospitalization and high mortality rates^7^, ^8^.

The significant spread of MDR *K. pneumoniae* all over the world has led to extensive use of carbapenem as last-resort antibiotics for treating the infections caused by those pathogens. Unfortunately, the emergence of carbapenem resistance among *K. pneumoniae* has been reported all over the world^7^. Among mechanisms of resistance, production of carbapenem hydrolyzing enzymes (carbapenemases) is considered as the most common mechanism of carbapenem resistance and a significant cause of worry in the Middle East and worldwide^9^–^13^.

Carbapenemases are β-lactamase enzymes belonging to classes A, B, C and D and can be categorized according to the dependency on divalent cations for the activation of the enzyme into metallo-carbapenemases, MBLs, (zinc-dependent class B) and non-metallo-carbapenemases (zinc-independent classes A, C, and D). The most commonly detected carbapenemases in Enterobacteriaceae are KPC (*Klebsiella pneumoniae* carbapenemase); GES (Guiana extended-spectrum); VIM (Verona integron encoded metallo-β-lactamase); IMP (imipenemase), NDM (New Delhi metallo-β-lactamase) and OXA-48 (oxacillinase-48)^14^.

Regarding the remarkable increase in carbapenem resistance among *K. pneumoniae* isolates, the world nowadays is concerned about screening for those carbapenemase-producing strains. In Egypt, there were several studies about carbapenem resistance, however, some information was known about carbapenem resistance in *K. pneumoniae* from Delta region. Therefore, this study was conducted to determine the incidence of carbapenem resistance-associated genes in CR-KP clinical isolates in Kafrelsheikh city, Egypt.

## Material and Methods

### Bacterial isolates

A total of 230 non-duplicated *K. pneumoniae* clinical isolates were recovered from 1005 different clinical samples that were collected during the period from July 2015 to April 2016 from hospitalized patients in different four hospitals in Kafrelsheikh city; Egypt. The *K. pneumoniae* isolates were recovered from urine (n = 98), blood (n =67), sputum (n = 55) and wound (n = 10).

### Bacterial identification

The collected isolates were identified according to Collee et al.^15^ using conventional standard biochemical tests including culturing on MacConkey agar, indole test, citrate utilization, urease test, oxidase reaction, methyl red test, and sugar fermentation. Besides, all isolates which were identified as *K. pneumoniae* were confirmed by the automated Vitek2® compact system (BioMérieux®). The recovered isolates were stored in 50.0 % glycerol stock at -80 °C for further analysis.

### Antimicrobial susceptibility testing

The Kirby-Bauer disk diffusion method was conducted for all 230 *K. pneumoniae* isolates using Mueller-Hinton agar and the results were recorded in accordance with CLSI 201516 guidelines. The used antimicrobial discs (Oxoid; UK) included beta-lactams as ampicillin (10µg), amoxicillin (10µg), amoxicillin/clavulanic acid (20/10µg), ampicillin/sulbactam (10/10µg), ticarcillin/clavulanic acid (75/10µg), piperacillin/tazobactam (100/10µg), meropenem (10µg), imipenem (10µg), cefoxitin (30µg), cefotetan (30µg), cefazolin (30µg), cefuroxime (30µg), ceftriaxone (30µg), cefotaxime (30µg), ceftazidime (30µg), cefepime (30µg), aztreonam (30µg); aminoglycosides as gentamicin (10µg), tobramycin (10µg), amikacin (30µg) and other antimicrobials including chloramphenicol (30µg), ciprofloxacin (5µg), tetracycline (30µg) and trimethoprim-sulfamethoxazole (1.25/23.75µg),. Susceptibility to tigecycline was detected using Vitek2® compact system (BioMérieux®). Minimum inhibitory concentrations (MICs) were detected for all CR-KP isolates using Vitek2® compact system (BioMérieux®). Any isolate would be recorded to have MDR profile if it showed non-susceptibility to ≥3 different antimicrobial classes^4^.

### Phenotypic detection of carbapenemases

All 50 CR-KP isolates were screened for carbapenemases production using MHT 16. MBLs were screened in all 50 CR-KP isolates using EDTA combined disc synergy test^17^, ^18^.

### Genotypic detection and characterization of carbapenemase genes by polymerase chain reaction and DNA sequencing

For genomic DNA extraction, GeneJET Genomic DNA Extraction Kit (Thermo Scientific, USA) was used. For all carbapenem non-susceptible isolates, PCR was performed to detect six of the most frequently widespread carbapenemase genes including blaKPC, blaGES, blaNDM-1, blaVIM, blaIMP and blaOXA-48.

The PCR technique was carried out, using the primers shown in Table 1, in a final volume of 50 µl consisting of 5 µl of DNA template (5–20 ng/ml); 25 µl of PCR master mix; 2.5 µl of forward and 2.5 µl of reverse primer (0.1–0.5 µM) and nuclease-free water (15 µl) was added to complete the final volume.

**Table 1:** Nucleotide sequences of PCR oligonucleotide primers

Target gene	Primer*	Sequence (5′ 3′)	Size (bp)	Reference
***bla***_KPC_	KPC-F KPC-R	CGTCTAGTTCTGCTGTCTTG CTTGTCATCCTTGTTAGGCG	798	Poirel, Laurent,2011^20^
***bla***_GES_	GES-F GES-R	AGTCGGCTAGACCGGAAAG TTTGTCCGTGCTCAGGAT	399	Dallenne, Caroline,2010^19^
***bla***_NDM-1_	NDM-F NDM-R	GGTTTGGCGATCTGGTTTTC CGGAATGGCTCATCACGATC	621	Poirel, Laurent,2011^20^
***bla***_VIM_	VIM-F VIM-R	GATGGTGTTTGGTCGCATA CGAATGCGCAGCACCAG	390	Poirel, Laurent,2011^20^
***bla***_IMP_	IMP-F IMP-R	GGAATAGAGTGGCTTAAYTCTC GGTTTAAYAAAACAACCACC	232	Poirel, Laurent,2011^20^
***bla***_OXA-48_	OXA-F OXA-R	GCGTGGTTAAGGATGAACAC CATCAAGTTCAACCCAACCG	438	Poirel, Laurent,2011^20^

* F: forward; R: reverse

The amplification was performed in the thermal cycler (Verti; Applied Biosystem) with initial denaturation for 10 min at 94°C. After denaturation, about 36 cycles of amplification were conducted including 30 s for denaturation at 94°C, 40 s for annealing at 52°C, and 50 s for extension at 72°C and the last step was the final elongation for 5 min at 72°C. DNA fragments were analyzed under UV light after 1 hour of agarose gel electrophoresis^19^,^20^.

The PCR Purification Kit (#PP-201S; Jena Bioscience, Germany) was used, according to the manufacturer's protocol, for purification of PCR products. For DNA sequencing, the purified PCR products were sent to Macrogen Company (South Korea) and sequenced using ABI 3730XL DNA sequencer (Applied Biosystem; USA). The analysis of obtained sequences was performed using the Chromas Lite 2.1 program, BLAST search was used to detect the identity of these sequences against Gen-Bank database and Geneious 4.8.4 software was used for alignments and assembly of the sequences.

## Results

The results of the standard biochemical tests, as well as the Vitek2® compact system revealed the presence of 230 *K. pneumoniae* isolates among the tested 1005 clinical samples.

The susceptibility testing results of the recovered 230 *K. pneumoniae* isolates revealed that the incidence of resistance among these isolates ranged from (0.0%) for tigecycline to (98.3%) for ampicillin. The results indicated that 21.7% of isolates were carbapenem non-susceptible where 50 isolates were meropenem resistant while for imipenem; 49 isolates were resistant and only one was intermediate resistant to such antimicrobial agent. On the other hand, 175 (76.0%), 161 (70.0%) and 138 (60.0%) isolates were resistant to ciprofloxacin, amikacin and trimethoprim/sulphamethoxazole respectively. Interestingly, all tested isolates were tigecycline sensitive. Moreover, all detected CR-KP isolates exhibited MDR profile.

Phenotypic detection of carbapenemases production was carried out using MHT and the results revealed that 45 (90.0%) out of 50 tested CR-KP isolates were carbapenemase producers. On the other hand, 33 (66.0%) out of 50 tested isolates were MBLs producers as detected by combined disk synergy test as shown in Table 2.

**Table 2:** Phenotypic and genotypic characterizations of tested carbapenem resistant *Klebsiella pneumoniae* isolates

Isolates	Sensitivity pattern*	Carbapenemases detection				

		Phenotypic results**		Genotypic results	

		MHT	CDST	*bla*_NDM-1_	*bla*_OXA-48_
K1	CN, SXT, TGC	+	-	_	_
K2	AK, SXT, TGC	+	-	_	_
K3, K6, K15, K16, K17, K22, K23, K24, K27, K32, K34, K35, K38, K42, K47, K49, K50	SXT, TGC	+	+	*bla*_NDM-1_	*bla*_OXA-48_
K4	CIP, TGC	+	-	_	*bla*_OXA-48_
K5	CIP, TGC	-	-	_	_
K7	CN, AK, CM, TET, CIP, SXT, TGC	-	+	*bla*_NDM-1_	*bla*_OXA-48_
K8	SXT, TGC	+	-	*bla*_NDM-1_	_
K9	CN, AK, TGC	+	+	*bla*_NDM-1_	_
K10, K11, K12, K14, K18, K20, K21	SXT, TGC	+	-	_	_
K13	CN, AK, CM, TET, CIP, TGC	+	-	_	_
K19	AK, CM, TET, CIP, SXT, TGC	+	+	*bla*_NDM-1_	_
K25	CN, AK, CM, TET, CIP, SXT, TGC	+	-	_	_
K26	AK, TET, CIP, SXT, TGC	-	+	*bla*_NDM-1_	*bla*_OXA-48_
K28, K40	SXT, TGC	+	+	*bla*_NDM-1_	_
K29	CN, AK, TET, CIP, TGC	+	+	*bla*_NDM-1_	_
K30	AK, TGC	+	-	_	_
K31	CN, AK, , CIP, TGC	+	+	*bla*_NDM-1_	*bla*_OXA-48_
K33	AK, TET, CIP, TGC	+	+	*bla*_NDM-1_	*bla*_OXA-48_
K36	TGC	+	-	*bla*_NDM-1_	*bla*_OXA-48_
K37, K39	CIP, TGC	+	+	*bla*_NDM-1_	_
K41	CN, AK, CM, TET, CIP, TGC	+	+	*bla*_NDM-1_	_
K43	CIP, TGC	+	+	*bla*_NDM-1_	*bla*_OXA-48_
K44	AK, CIP, SXT, TGC	+	+	*bla*_NDM-1_	*bla*_OXA-48_
K45	SXT, TGC	-	-	_	*bla*_OXA-48_
K46	AK, SXT, TGC	+	+	*bla*_NDM-1_	_
K48	AK, CIP, SXT, TGC	-	+	*bla*_NDM-1_	_

All CR-KP isolates were screened by PCR for the detection of blaKPC, blaGES, blaNDM-1, blaVIM, blaIMP and blaOXA-48 genes. The results revealed that blaNDM-1 and blaOXA-48 were the only detected genes where blaNDM-1 was detected in 35 (70.0%) isolates while blaOXA-48 was detected in 26 (52.0%) isolates. Besides, up to 37 (74.0%) isolates carried at least one of the recorded genes where 24 isolates harbored both detected genes while 13 isolates harbored only one of these genes as shown in Table 2.

## Discussion

The incidence of CR-KP isolates in the Mediterranean area particularly in Egypt represents a serious threat to our hospitals and our community^21^. Therefore, this study was performed to detect the prevalence of carbapenemase genes among *K. pneumoniae* isolates recovered from Egyptian patients in different hospitals in Kafrelsheikh city, Egypt. Our results revealed that the incidence of *K. pneumoniae* isolates among different clinical samples was 22.8%. Susceptibility testing results showed that up to 21.7% of the tested *K. pneumoniae* isolates were carbapenem non-susceptible. In agreement with our result, previous studies from Egypt^22^ and different countries^23^ showed comparable results.

The antimicrobial susceptibility testing results of CR-KP isolates showed a high incidence of MDR profile where all tested isolates exhibited multiple antimicrobial resistance, a finding that was also reported in other studies from different countries^24^–^27^. This finding might reflect unrestricted use of antibiotics in our institute which plays an important role in increasing antibiotic resistance. It worth mentioning that tigecycline was the most active antimicrobial agent where all tested isolates were sensitive to it. This result was confirmed by the results of other studies^28^–^30^ which also reported 100% sensitivity of the isolates to tigecycline.

In the present study, the detection of carbapenemase producers was carried out using phenotypic and genotypic methods. Regarding the phenotypic methods, our results revealed that 90.0% of CR-KP isolates were carbapenemase producers. Moreover, the results revealed that 66.0% of tested isolates were MBLs producers and this result was comparable (63.3%) with that detected by Panchal et al.^23^ but in contrast, lower (41.9%) result was detected in another study which was performed in Saudi Arabia^31^.

The results of genotypic detection methods revealed that 74.0% (37/50) of CR-KP isolates were harboring to carbapenemase genes which was higher than that reported in other Egyptian study^32^ where carbapenemase genes were detected in only 43.0% *K. pneumoniae* isolates. Interestingly, out of the 37 isolates carrying carbapenemase genes that detected by PCR, there were 4 isolates that showed negative results by MHT and this might be explained by weak carbapenemase activity^33^. On the other hand, 13 (26.0%) of the CR-KP isolates were negative for carbapenemase genes. These isolates may contain other untested carbapenemases or other mechanisms such as extended-spectrum beta-lactamase production coupled with disruption in porin expression may be responsible for their reduced sensitivity to carbapenems^34^–^36^.

Enterobacteriaceae particularly *K. pneumoniae* producing blaOXA-48 and blaNDM-1 became more prevalent in diverse of the world. In our study, blaOXA-48 and blaNDM-1 were the only detected carbapenemase genes among the tested *K. pneumoniae* isolates. This result was in agreement with other performed studies in Egypt^36^, ^37^; revealing the emergence of the dissemination and rapid spread of these genes through different regions in Egypt. In addition, our result was also reported in other countries 38, 39. Since the first identification of blaNDM-1 from *K. pneumoniae* retrieved from a Swedish patient of Indian origin traveled to New Delhi, India 40, worldwide attention was attracted due to the rapid dissemination of the MBLs. In Egypt, the first isolation of blaNDM-1 was reported by Abdelaziz et al.^41^ from ST11 *K. pneumoniae* isolate that was recovered from a hospitalized patient in an ICU of a cancer hospital in Cairo and after that blaNDM-1 was described in *Pseudomonas aeruginosa* isolates by Zafer et al.^42^ and in *Acinetobacter baumannii* isolates by El-Sayed et al.^43^ revealing the spread of blaNDM-1 among *Enterobacteriaceae* particularly *K. pneumoniae* isolates^44^. In the present study, blaNDM-1 was identified as a predominant gene and detected in 70.0% of the tested isolates. This finding was consistent with other studies that recorded the predominance of blaNDM-1 in Egypt and other countries^45^–^49^.

Regarding the blaOXA-48 gene, it was first detected in K. pneumoniae in Turkey^50^ and after that, it spread rapidly throughout the Middle East and then all over the world. In the present study, our results revealed that the second most common carbapenemase gene detected among tested isolates was blaOXA-48 with an incidence of 52.0%. In accordance with our result, Khalifa et al.^36^ and ELMahallawy et al.^37^ from Egypt reported comparable (49.0% and 60.0%, respectively) detection rate of blaOXA-48 among carbapenem-resistant isolates. In other Arabian countries, this detected gene was also reported as one of the most detected carbapenemases genes with high incidence such as 66.0% incidence in Riyadh^31^ and 62.0% incidence in Lebanon^51^.

Carbapenem-resistant isolates co-producing multiple carbapenemases genes tend to be extremely high resistant and this leads to limitation in treatment options. Our study showed that 48.0% of tested CR-KP isolates harbored both blaOXA-48 and blaNDM-1, a finding that is considered as the first report of a high incidence of blaOXA-48 and blaNDM-1 co-existence among CR-KP isolates in Egypt. In agreement with our detection rate, Laolerd et al.^52^ reported also 52.0% as an incidence of isolates that also harbored those both genes. In contrast, neither blaKPC, blaGES, blaVIM nor blaIMP was detected in any of our tested isolates. Other studies also showed very low detection rates of these genes as EL-Mahallawy et al.^37^ who reported that the incidence of blaIMP was only 1.5% with the absence of blaKPC and blaVIM among the tested isolates. Additionally, Khalifa et al.^36^ reported a low (4.6 %) incidence rate of blaVIM with the absence of blaKPC and blaIMP. In accordance with our results, Al-Agamy et al.^31^ also reported the absence of blaIMP, blaVIM, blaGES and blaKPC in the carbapenemase-positive isolates confirming that those genes are not predominate in our geographical region.

## Conclusion

This study reported a high MDR resistance profile among the CR-KP isolates from Kafrelsheikh, Egypt. This high resistance rate in our study may be attributed to the lack of implementation of antimicrobial stewardship program and this reflects also limiting in treatment options. In addition, our study reported the emergence of K. pneumoniae isolates co-harboring blaNDM-1 and blaOXA-48 genes. These findings may be considered as an alarming threat to the healthcare workers in the hospital settings. The results of our study therefore confirmed the need for continually and routinely screening for K. pneumoniae strains especially those producing blaNDM-1 and blaOXA-48 genes to rapidly detect and avoid the dissemination of those strains.
